# “Time Is Brain” – for Cell Therapies

**DOI:** 10.1002/advs.202519579

**Published:** 2025-11-18

**Authors:** Hao Yin, Dominikus Brian, Rebecca Z. Weber, Patrick D. Lyden, Ruslan Rust

**Affiliations:** ^1^ Robarts Research Institute Schulich School of Medicine and Dentistry Western University London Ontario N6A 5C1 Canada; ^2^ OrganoBit BioIntelligence Pte Ltd Singapore 049422 Singapore; ^3^ Department of Physiology and Neuroscience University of Southern California Los Angeles CA 90033 USA; ^4^ Zilkha Neurogenetic Institute Keck School of Medicine University of Southern California Los Angeles CA 90033 USA

**Keywords:** cell therapy, cell transplantation, neural stem cells, stroke

## Abstract

The principle “time is brain” has long guided acute stroke treatment, emphasizing that earlier intervention improves outcomes. While this dictum applies to current gold‐standard reperfusion therapies, its relevance to emerging regenerative approaches such as stem cell therapy remains to be established. A growing body of preclinical and clinical studies suggests that timing of cell delivery is a key determinant of graft survival, integration and therapeutic efficacy, largely through interactions with the evolving post‐stroke microenvironment. Here, we discuss how early transplantation may access salvageable tissue but faces a hostile inflammatory microenvironment, whereas transplantation at the subacute or chronic phase benefits from a more permissive milieu but by then much of the tissue has been irreversibly lost. We further suggest the optimal window also depends on cell type and mechanism of action: neuroprotective or immunomodulatory grafts may benefit from earlier delivery, while cells requiring long‐term survival and integration may perform better later. Thus, “time is brain” also applies to cell therapies, but it may require aligning graft delivery with the evolving post‐stroke microenvironment rather than the acute therapeutic window. Identifying biomarkers that track inflammatory changes, vascular remodeling and brain damage could personalize this “window of receptivity” and guide tailored future clinical trials.

## Graphical abstract

The concept that “time is brain” extends to stem cell therapy for stroke. Evidence across preclinical and clinical studies indicates that delivery timing shapes graft survival, integration, and efficacy by matching the evolving post‐stroke microenvironment. Biomarkers and imaging may personalize a receptive window and guide future cell therapy trials.

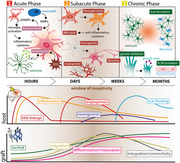

## 1 Introduction

The principle of “time is brain” has long guided management of acute ischemic stroke. On average, a patient with a large vessel stroke can lose 1.9 million neurons, 14 billion synapses, and 12 km of myelinated fibers each minute without treatment.^[^ ^1^, ^2^ ^]^ Accordingly, current reperfusion therapies (thrombolysis and thrombectomy) are most effective when applied as soon as possible after the last known well time (LKWT). While guidelines limit the LKWT to up to 4.5 h for intravenous thrombolysis and, in carefully selected patients, up to 24 h for mechanical thrombectomy, these limits are changing as advanced neuroimaging gains wider acceptance.^[^ ^3^, ^4^, ^5^, ^6^, ^7^ ^]^ While acute reperfusion therapies can be highly effective, many patients cannot receive them due to delayed hospital arrival, contraindications such as increased hemorrhagic risk, or limited access to comprehensive stroke centers. As a result half of the stroke survivors, 3 million people each year, are left with enduring and disabling motor, language, or cognitive deficits.^[^ ^8^ ^]^ These patients have only limited treatment options besides rehabilitation and could benefit from regenerative therapies that restore lost brain function.

Leading experts in the Stroke Treatment Academic Industry Roundtable (STAIR) recognize stem cell‐based therapies as one of the most promising regenerative approaches to improve stroke outcomes.^[^ ^9^ ^]^ Similarly, Stem Cell Therapies as an Emerging Paradigm in Stroke (STEPS) has issued guidelines for preclinical studies of stem cell therapy for stroke.^[^ ^10^ ^]^ Yet, unlike thrombolysis or thrombectomy, which act within hours, the therapeutic potential of regenerative stem cells operates on a different timeline. The success depends on the ability of grafted cells to survive, migrate, differentiate, and/or integrate into a highly dynamic post‐stroke tissue environment; processes that evolve over days to weeks rather than minutes to hours. Nonetheless, both acute and regenerative strategies share a key principle: the timing of intervention critically determines therapeutic efficacy and long‐term outcome. Recognizing and defining this “window of receptivity” for cell transplantation after stroke may prove just as essential for regenerative therapies as the discovery of the limited time from LKW therapeutic window for recanalization therapies, guiding both preclinical design and future clinical translation.

In this review, we describe how transplantation timing influences the efficacy of cell‐based therapies for stroke. We discuss evidence from preclinical and clinical studies, providing a comprehensive overview of how variations in timing have affected outcomes across rodent models and stroke patients. We further propose that a reproducible “window of receptivity” may exist between the acute and chronic phases and suggest that emerging biomarkers could help guide the next generation of cell therapies for stroke and potentially related neurological disorders.

## 2 The Challenge of Timing a Cell Therapy for Brain Repair

An ischemic stroke triggers an acute and severe disruption of the cerebral microenvironment, posing multi‐level challenges for the timing of cell transplantation. Within hours, primary ischemic tissue damage leads to widespread local and systemic inflammatory responses, characterized by activation of microglia, and infiltration of neutrophils, monocytes, and lymphocytes.^[^ ^11^ ^]^ These processes drive a surge of cytokines and reactive oxygen species, as well as blood‐brain barrier (BBB) breakdown that facilitates further infiltration of peripheral immune cells and exacerbates secondary tissue damage.^[^ ^11^, ^12^ ^]^ This post‐stroke environment not only accelerates neuronal loss but also establishes a microenvironment unfavorable to transplanted therapeutic cells, severely limiting graft survival, a so‐called hostile environment. Cell transplantation at chronic stages after stroke (>6 months) may favor graft survival as inflammation subsides, however, by this time cells within the ischemic border zone, defined as the metabolically compromised but salvageable tissue surrounding the infarct core, have largely disappeared. Instead, the cavity becomes occupied by glial scar, axon growth‐inhibitory proteins, and extracellular matrix, significantly reducing brain plasticity.^[^ ^13^, ^14^, ^15^, ^16^, ^17^ ^]^

Between these two extremes is the subacute phase, ranging from 3 days to a few weeks, when the post‐stroke brain shifts from a pro‐inflammatory, tissue‐degrading mode into a reparative mode. For example, microglia transition from an early debris‐cleaning and DAMP‐releasing phenotype to an anti‐inflammatory, reparative phenotype that promotes neurogenesis and remyelination via secreting neurotrophic factors (e.g., TGF‐β, IL‐4, IGF‐1, IL‐10, BDNF, and galectin‐9).^[^ ^18^, ^19^, ^20^, ^21^ ^]^ In parallel, the microvasculature undergoes active angiogenic remodeling during the early subacute phase (days 3–7), which is likely beneficial,^[^ ^16^, ^22^, ^23^ ^]^ The growing microvessels provide oxygen, nutrients, and even potential neuron guidance cues in the peri‐lesional zones, supporting the survival, migration, and differentiation of neural stem cells.^[^ ^16^, ^24^, ^25^ ^]^ However, newly formed blood vessels may also exhibit increased permeability, facilitating infiltration of pro‐inflammatory macrophages that exacerbate parenchymal injury.^[^ ^26^ ^]^ Considering these multifaceted changes, whether the subacute phase represents a true “window of receptivity” remains uncertain.

The uncertainty surrounding optimal timing of cell therapy, ranging from 1 day to 1 month post‐injury (e.g., 1, 4, 7, or 30 days), had already existed since the earliest landmark studies.^[^ ^27^, ^28^, ^29^, ^30^, ^31^, ^32^ ^]^ Thus, the central question is not only whether cells should be delivered early or late, but whether there is a temporally defined window in which the host brain is most receptive, a possibility now being explored in preclinical studies. In a broader context, the optimal timing of the course also depends on other factors including the cell type transplanted, stroke model, delivery methods, and the therapeutic benefits aimed, as described in more detail elsewhere.^[^ ^33^ ^]^

Taken together, these observations highlight that cell transplantation timing is complex. We propose that matching cell transplantation timing to the dynamic, evolving post‐stroke environment will likely yield successful graft survival and integration with improved beneficial effects on neurological recovery.

## 3 Preclinical Evidence on Timing of Cell Therapies for Stroke

**Figure 1:**
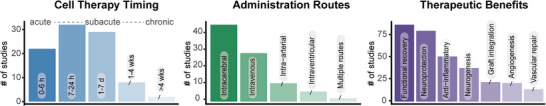
Summary of transplantation timing, administration routes, and reported therapeutic benefits of stem cells across preclinical stroke studies. Neuroprotection refers to the protection of host neurons specifically, instead of transplanted stem cells, endogenous glia, or neurovascular units.

Recent preclinical studies that treated transplantation timing as a central variable have shown that delaying cell delivery into the subacute phase often enhances graft survival, proliferation, and integration into host circuits. This pattern has been observed across multiple cell types and injury models, though benefits have also been reported with acute or chronic delivery. In our analysis of preclinical cell therapy studies for stroke (**Figure** **1**; Table S1, Supporting Information), the majority delivered cells in the acute (7–24 h) or subacute (1–7 days) windows, which together accounted for more than 60% of studies, while hyperacute (0–6 h) and chronic treatments (>4 weeks) were less common. It should be noted that ≈85% of these studies performed cell transplantation at only a single time point. Therefore, this predominance likely reflects common experimental designs that produce observable benefits, rather than definitive evidence of the best treatment window. Across administration routes, intracerebral injection was the most frequent (≈40% of studies), followed by intravenous delivery and fewer intra‐arterial or intraventricular deliveries. The reported therapeutic benefits include neurofunctional recovery and histological evidence for repair and regeneration of neural and vascular tissues. Functional recovery and neuroprotection were the most consistently reported benefits, while additional effects such as anti‐inflammatory responses, neurogenesis, and angiogenesis appeared less frequently (Figure 1; Table S1, Supporting Information).

**Figure 2:**
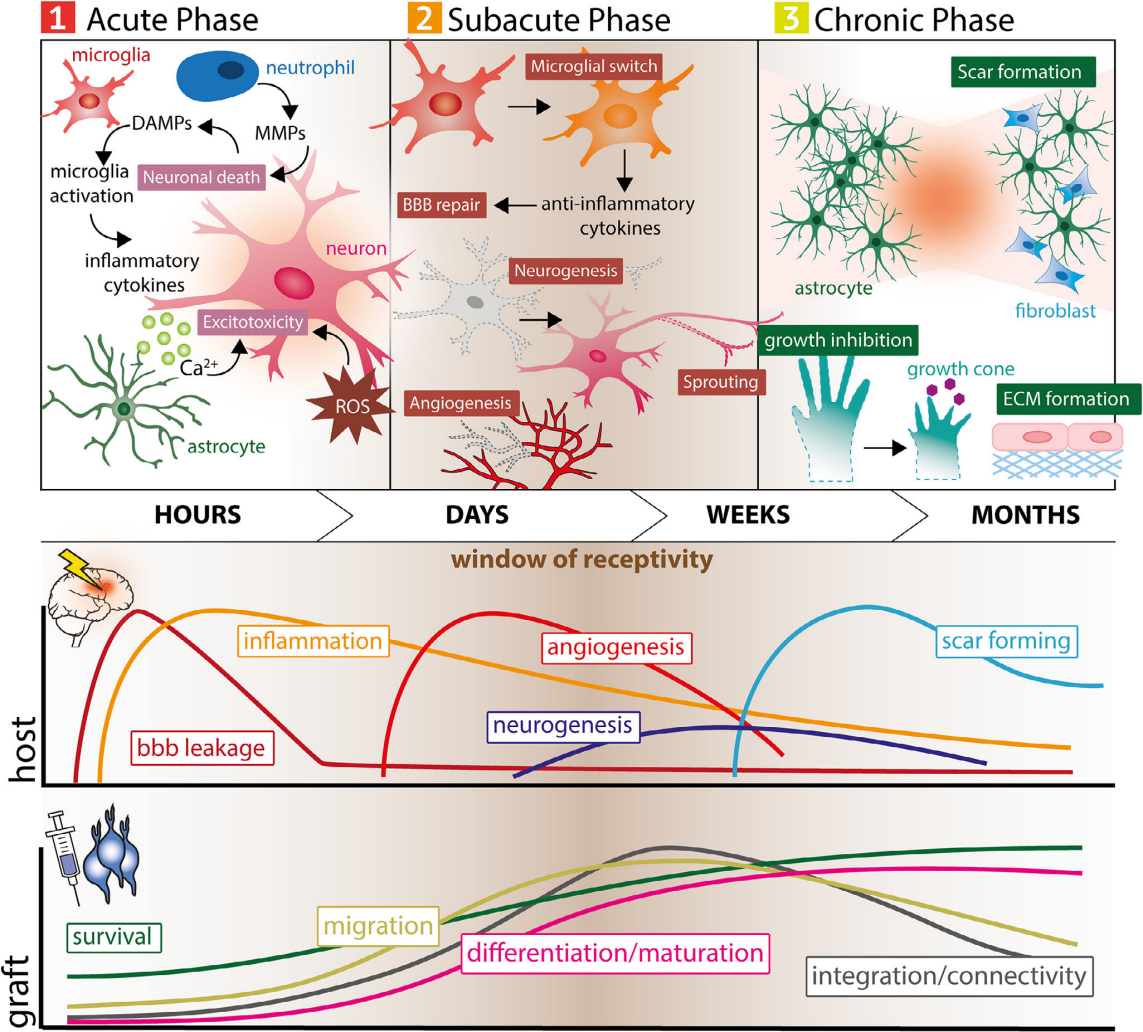
Evolving stroke environment for cell grafts in the acute, subacute, and chronic phase. In the acute phase, inflammation and excitotoxicity create a hostile environment with limited graft survival and integration. The subacute phase is marked by reduced inflammation, BBB repair, and angiogenesis, offering a permissive window with improved survival and integration potential. In the chronic phase, scar and extracellular matrix (ECM formation limit integration, though survival remains possible.

The optimal window appears to depend not only on properties of the graft but, perhaps equally important, on the evolving host environment (Figures 1 and **2**). Below, we discuss representative studies, providing the most recent overview of diverse experimental designs and associated cellular and tissue outcomes.

In a mouse model of photothrombosis‐induced cortical stroke, delayed stereotactic injection of neural precursor cells (NPCs) differentiated from human induced pluripotent stem cells (hiPSCs) at 1 day vs 7 days post‐injury was compared.^[^ ^34^ ^]^ Longitudinal bioluminescence imaging revealed a fivefold higher signal over six weeks when NPCs were delivered on day 7, indicating superior survival or proliferation of the grafted cells compared to acute delivery. Mechanistically, graft proliferation was almost doubled with delayed delivery (15.1% vs 7.6% 5 ethynyl 2 deoxyuridine(EdU) incorporation, indicating the fraction of grafted cells that were actively dividing), with similar differentiation potentials (neuronal vs astrocytic fate) across different time points. Moreover, 40% of the delayed grafts extended their neurites along the corpus callosum to the contralateral projections, which was not observed for acute grafts. Notably, although delayed transplantation did not significantly modify GFAP + astroglial scarring or apoptosis in the ischemic core or border zone by 6 weeks, the derived GABAergic neurons communicated with injured host tissue via SLIT and NRG signaling to promote neuronal regeneration/projection, dendritic arborization, synaptic plasticity and BBB integrity, leading to long‐term functional recovery (gait pattern and locomotor skills) of stroke mice.^[^ ^35^ ^]^

Importantly, post‐stroke benefits of cell transplantation at the subacute phase have been reported by other independent groups. In earlier work, intravenous delivery of adult syngeneic NPCs at 72 h post‐injury confers neuroprotective effects in a mouse stroke model induced by transient middle cerebral artery occlusion.^[^ ^36^ ^]^ Mice receiving NPCs displayed enhanced recovery of motor skills and stronger grip strength, correlated with a 20% reduction in lesion volume by 30 days and histological improvement of inflammation, astrogliosis, and neuronal survival. Estimated, only 0.3% of injected NPCs were found home to ischemic border zone, among which less than 5% were immunoreactive of neuronal lineage markers, suggesting an undifferentiated status of injected NPCs, even successfully localized at injured regions. Similarly, in a mouse model of traumatic motor cortical injury, a 7‐day delay between lesion and transplantation of embryonic cortical neurons yielded a significant benefit over graft survival, proliferation, vascularization, neuronal projections, and neurobehavioral performance, as compared with immediate transplants.^[^ ^37^ ^]^ Of note, 7 days, as well as other subacute time points (3–14 days) have been reported as an experimental selection in a handful of animal studies involving a wide range of stem/progenitor cells, including bone marrow‐ or adipose‐derived mesenchymal stem cells (MSCs), hPSC‐derived, or primary neural stem/progenitor cells (NSCs/NPCs), hPSC‐derived glia‐enriched progenitors, and even hPSC‐derived cerebral organoid.^[^ ^38^, ^39^, ^40^, ^41^, ^42^, ^43^, ^44^, ^45^ ^]^ However, preclinical benefits have also been achieved for a similar spectrum of cells when injected at acute^[^ ^46^, ^47^, ^48^ ^]^ or chronic time points.^[^ ^49^, ^50^, ^51^ ^]^

It must be emphasized that there are only a few studies that systematically evaluate the impact of transplantation timing on the efficacy of cell therapy, and most of them compared different time points within the acute phase (i.e., the first 48 h).^[^ ^52^, ^53^, ^54^, ^55^, ^56^ ^]^ Within the limited available studies spanning acute and subacute studies, differences in cell types, route of delivery, and stroke models greatly confound the interpretation, making a consensus in the selection of optimal timing impractical. One of the earliest cell therapy studies demonstrated that intravenous delivery of bone marrow MSCs at both 1‐ and 7‐day post‐stroke resulted in significant recovery of somatosensory behaviors and neurological severity score in rats.^[^ ^30^ ^]^ Moreover, intra‐carotid arterial delivery of endothelial progenitor cells was conducted at 3‐h, 3‐, 7‐, 14‐, or 28‐day post‐stroke in rats. While 3‐h and 3‐day delivery exhibited similar benefits in infarct volume and neurological functions, such benefits progressively diminished with prolonged delay of transplantation.^[^ ^57^ ^]^ In terms of therapeutic timing, bone marrow‐derived mononuclear cells (BM‐MNCs), which contain mixed populations of stem/progenitor cells and are also tested in human trials, also offer an important line of evidence. Intravenous delivery of autologous BM‐MNCs at 3–72 h after induction MCAO in rats revealed the strongest histological protection within the hyperacute phase (3 or 6 h), but reduction in lesion size was still evident with cells delivered at 72 h.^[^ ^58^ ^]^ In spontaneously hypertensive rats with permanent MCAO, intravenous administration of human umbilical cord blood MNCs at 4, 24, or 72 h post‐stroke led to a significant recovery of sensorimotor functions with reduced infarct volume, brain atrophy, and glial scarring, while cell treatment at 5 or 14 days exhibited diminished or no benefits.^[^ ^59^ ^]^ In rats with a tandem left common carotid artery and left MCAO, dose‐ and time‐dependent intravenous delivery of autologous BM‐MNCs defined a therapeutic window of 72 h post‐stroke with 10 million cells per kilogram, based on neurobehavioral tests and histological lesion size. As a comparison, cell grafts administrated at 7 days showed no benefits. Of note, the injected cells can be observed at peri‐infarct regions and peripheral organs between 1 h and 7 days, suggesting a paracrine mechanism.^[^ ^60^ ^]^ In summary, these documented discrepancies are likely a reflection of the diversified inconsistency of cells and stroke models, and also a lack of mechanistic understanding of how different cell types engraft and communicate with the stroke‐injured brain niche.

MSCs and BM‐MNCs have been reported to lack the capacity of neural lineage commitment in vivo.^[^ ^61^, ^62^ ^]^ Instead, they are considered to support post‐stroke brain repair via neurotropic and paracrine actions and immunomodulation. While their homing to the ischemic site is usually minimal (<1% of injected cells) and short‐term, the long‐term engraftment is likely not required for their benefits. Thus, transplanted MSCs or BM‐MNCs are often delivered intravascularly in the acute or subacute phase, aligning with their role as an immune/inflammation regulator, instead of cell replacement. On the other hand, NSCs/NPCs exhibit high potential of neural and glial differentiation and are able to functionally integrate into regenerating neural circuits.^[^ ^63^, ^64^, ^65^ ^]^ Furthermore, NSCs/NPCs can release growth factors to support endogenous neurogenesis, angiogenesis, and regain of neurovascular homeostasis. As NSCs/NPCs must survive and mature in the host environment to replace the damaged tissue, optimal transplantation would likely be during the subacute phase to avoid direct confrontation with a destructive inflammatory environment (even though NSCs/NPCs are reported to possess modest immunomodulatory property^[^ ^66^, ^67^ ^]^). Similar to NSCs/NPCs, hiPSC‐derived glia‐enriched progenitors fit into a subacute window, since they need to differentiate into astrocytes predominantly at local injury sites to form a neurotrophic astroglial network that supports remyelination and axonal growth.^[^ ^44^, ^68^ ^]^ Notably, glia‐enriched progenitors have minimal neuronal output, and likely do not require indefinite survival. Therefore, even transplantation at the chronic phase may help to increase the plasticity and stability of regenerated brain tissue. To resolve this complexity and these context‐dependent discrepancies, we need studies that place transplantation timing at the core of their design. Although some of the studies discussed provide valuable insights, broader and more systematic comparisons will be required to define clinically relevant therapeutic windows for different cell types and delivery routes.

Importantly, timing cell therapies is not a challenge unique to stroke. Related preclinical studies have demonstrated the therapeutic potential of stem and progenitor cell therapy in a variety of neurological disorders including brain aging,^[^ ^69^, ^70^ ^]^ Alzheimer's disease,^[^ ^69^, ^71^ ^]^ Parkinson's disease,^[^ ^72^, ^73^ ^]^ and traumatic brain injury.^[^ ^74^, ^75^ ^]^ Across these diverse conditions, the temporal dynamics of transplantation emerge as a recurring determinant of graft survival, integration, and functional benefit. These findings suggest that the notion of a “therapeutic window” is broadly relevant beyond stroke, although its exact boundaries likely vary by disease mechanism and host environment. In parallel, recent advances that use biochemical and biophysical cues to enhance the survival and engraftment of transplanted cells^[^ ^76^, ^77^, ^78^, ^79^ ^]^ represent another promising and rapidly growing direction for cell transplantation research, which lies beyond the scope of this review.

## 4 Can Biomarker Define the Therapeutic Window for Cell Therapy After Stroke?

Most biomarker research in stroke has aimed at predicting recovery or guiding acute interventions. For example, blood‐based biomarkers can provide a cost‐effective, dynamic picture of pathophysiologic phases, and neuroimaging can facilitate the detection of the location and severity of the infarct core and salvageable border zone. Both modalities could also have the potential to guide the therapeutic window selection for cell therapy.

In the acute period (within 24 h of stroke onset), neurovascular inflammation, BBB breakdown, and coagulation induce elevation of MMP‑9, TNF‑α, IL‐6, VCAM‑1/ICAM‑1, E‐/P‐/L‐selectins, D‐dimer, and glial and neuronal damage markers such as GFAP, S100, S100B, and neuron‐specific enolase.^[^ ^80^, ^81^, ^82^ ^]^ Cardiac peptides (Brain Natriuretic Peptide/BNP, NT‑proBNP) also rise due to stroke‐related heart dysfunction.^[^ ^80^, ^83^, ^84^ ^]^ These markers, measured within 24 h for diagnosis of stroke, can delineate a pro‐inflammatory, hostile microenvironment unsuitable for neurorestorative cell therapy.

During the subacute phase, circulatory factors emerge to reflect the vascular remodeling, inflammation resolution, and persistent neuronal damage/repair, including MMP9/TIMP1 ratio, VEGF‐A, and neurofilament light chain (NFL) (Table S2, Supporting Information).^[^ ^85^, ^86^, ^87^ ^]^ The MMP9/TIMP‐1 ratio, surrogating active matrix degradation activity, has recently been reported to inversely correlate with improvements in depressive symptoms and cognitive functions during early rehabilitation in stroke patients.^[^ ^85^ ^]^ This may be attributed to the dose‐dependent effect of MMP9 activity: excess MMP9 damages the neural and vascular tissue compartments, while moderate activity facilitates neuroplasticity and angiogenesis.^[^ ^88^, ^89^ ^]^ In this regard, a declining MMP9/TIMP‐1 ratio suggests a shift from destructive to reparative phases, which may be amiable for cell transplantation. At a similar time period, the NFL, as a marker of ongoing axonal injury, appears to be the most robust subacute biomarker that can predict stroke outcomes and complications. Its plasma level elevates between 9‐ and 21‐days following stroke, within the subacute phase (1 week to 3 months) of human stroke.^[^ ^87^, ^90^, ^91^, ^92^ ^]^ Notably, across all major types of stroke, elevated NFL plasma level correlates with infarct volume, and predicts poor functional recovery at 3 and 6 months and all‐cause mortality in 3 years following stroke.^[^ ^87^, ^92^ ^]^ This temporally restricted release of NFL in the circulation likely captures persistent neuronal damage and may be repurposed to estimate the personalized therapeutic window for cell transplantation. Moreover, plasma exosomal microRNA content displays phase‐dependent variation. While miR‐30a‐5p peaks within the hyperacute phase (6 h), miR‐21‐5p level arise during subacute phases (8–14 days), respectively.^[^ ^93^ ^]^ Thus, MMP9/TIMP‐1 ratio, NFL level, and miR‐30a‐5p/miR‐21‐5p levels may be combined to mark the injury‐to‐repair transition of stroke brain and guide the personalized timing for the neurorestorative cell therapy.

Moreover, advanced neuroimaging enables quantitative assessment of both structural and functional aspects of the stroke brain, which helps to distinguish the subacute phase (Table S3, Supporting Information).^[^ ^94^ ^]^ Magnetic resonance imaging (MRI) provides both anatomical and biophysical insights. Acute infarcts show limited diffusion, attributable to cytotoxic edema, and appear hyperintense on diffusion‐weighted MRI (DWI), and hypointense on apparent diffusion coefficient (ADC) maps. During the early subacute phase (7–10 days), the ADC value starts to normalize with the increase in extracellular edema and consequent water movement, while DWI may still remain hyperintense.^[^ ^95^, ^96^, ^97^ ^]^ These MRI alterations mark a transition from the acute to subacute phase, when inflammation and regeneration co‐exist. Other MRI contrast modes allow characterization of a wide spectrum of brain structural and functional features. For example, T2/FLAIR hyperintensity signifies vasogenic edema, MR perfusion assesses regionalized cerebral blood flow, and functional MRI or MR spectroscopy detects the dynamics of neurovascular coupling and brain metabolism.^[^ ^98^, ^99^ ^]^ Furthermore, CT angiography and CT perfusion can provide rapid and critical geographic and hemodynamic information. CT angiography identifies the arterial blockage and collateral network, and CT perfusion delineates the infarct zone and ischemic border zone area,^[^ ^100^, ^101^ ^]^ guiding cell transplant locations. Extending beyond the conventional imaging modalities, positron emission tomography (PET) and other molecular imaging techniques enable the visualization of functional and inflammatory states of the stroke brain. For example, [^18^F]PET detects the signals of the glucose metabolic flux and bioenergetic state in different zones of the stroke brain, with a decrease and an increase in ^18^F‐fluoro‐2‐deoxy‐D‐glucose uptake consistently observed for ischemic core and peri‐infarct zone, respectively. TSPO, 18 kD mitochondrial translocator protein, is elevated in activated microglia in stroke and other CNS pathologies, making the TSPO ligand one of the most widely used PET tracer family (e.g., ^11^C‐PK11195, ^11^C‐PBR28, ^18^F‐DPA‐714, ^18^F‐GE‐180) to monitor neuroinflammation.^[^ ^102^, ^103^, ^104^ ^]^

In sum, given the highly variable disease severity and recovery course among individual stroke patients, we proposed that repurposing a combination of blood‐ and imaging‐based multimodal biomarkers may help to personalize the optimal therapeutic window for cell therapy. Beyond timing, these biomarkers enable continuous monitoring of cell therapy activities, and neuroimaging is particularly suited to quantify therapeutic impacts on brain microarchitecture, perfusion, BBB integrity, and neuroinflammation. Prospective biomarker‐guided studies are needed to validate if such strategies would improve patient outcomes with stem cell therapies.

## 5 Clinical Evidence on Timing of Cell Therapies for Stroke

Most clinical studies also emphasize that timing is crucial for cell therapy in stroke (Table S4, Supporting Information). Early clinical cell therapy trials have mostly been conducted in the chronic phase after stroke, often 6 months to several years after stroke using either fetal or adult stem cells.^[^ ^105^, ^106^, ^107^, ^108^, ^109^ ^]^ These studies were considered clinically more feasible and focused primarily on safety with some also reporting signals of functional benefits including reduced disability and enhanced activities of daily living.^[^ ^105^, ^106^, ^107^, ^108^, ^109^ ^]^ Delayed transplantations have practical advantages, including greater patient stability, lower risk of hemorrhagic transformation or edema, and clearer lesion assessment, and it can be combined with rehabilitation. At this stage, however, scarring and tissue loss are more advanced, making regeneration less likely. While some initial trials reported encouraging effects, the largest chronic stroke trial to date was the sham‐controlled Phase 2b ACTIsSIMA study (n = 163, NCT02448641). In this study, patients 6–60 months after stroke (median ≈22 months) underwent stereotactic implantation of SB623 cells, which are allogeneic mesenchymal stem cells transiently modified to express the Notch‐1 intracellular domain. The primary outcome was motor recovery, measured by the Fugl‐Meyer motor score, which assesses motor function after stroke. The trial did not meet its prespecified endpoint of a ≥10‐point improvement in Fugl‐Meyer total score at 6 months compared with sham surgery.^[^ ^110^ ^]^ Exploratory subgroup analyses, such as in patients with smaller infarcts, suggested a possible benefit.^[^ ^110^ ^]^

In contrast, more recent efforts have also moved into the acute and subacute windows, aiming to take advantage of neuroprotective and plasticity‐promoting mechanisms before irreversible scarring occurs. In the largest trials, intravenous administration of multipotent adult progenitor cells in the MASTERS^[^ ^111^ ^]^ (n = 129, NCT01436487) and TREASURE^[^ ^112^ ^]^ (n = 206, NCT02961504) trials showed acceptable safety, but neither achieved their prespecified efficacy endpoints within 90 days. Both studies, however, generated hypotheses that even earlier intervention and better patient selection may be critical, directions now being pursued in MASTERS‐2 (*n* = 300, planned, NCT03545607).

Another limitation of these earlier trials is the poor delivery efficiency typically associated with the intravenous route, which remains the preferred clinical approach due to its safety in acute stroke patients. While the intra‐arterial route carries the risk of cerebral embolisms or hemorrhage,^[^ ^113^, ^114^ ^]^ the combinatorial application of advanced microcatheters and imaging technologies may mitigate the risks and off‐target effects.^[^ ^115^, ^116^ ^]^ Earlier therapies may also be beneficial when paired with rehabilitation. Preclinical studies indicate that the timing of regenerative interventions relative to rehabilitation is critical, with evidence suggesting that receiving therapy first and subsequently combining it with rehabilitation can enhance functional outcomes compared to starting both at the same time.^[^ ^117^, ^118^ ^]^

Importantly, most completed clinical trials have tested adult primary stem cell therapies, predominantly MSC‐ or HSC/progenitor‐based approaches, which together account for over 90% of the clinical studies (Table S4, Supporting Information). Therefore, these interpretations largely reflect to the therapeutic window of MSC, where benefits are thought to arise primarily through indirect neuroprotective and immunomodulatory effects rather than direct tissue replacement.^[^ ^11^, ^119^ ^]^

For NSC‐based therapies, the optimal therapeutic window remains largely unknown. NSCs have only been tested in a small fraction of trials, most prominent PISCES‐I‐III. PISCES‐I and II demonstrated safety and exploratory signals of motor improvement following intracerebral transplantation of the CTX0E03 neural stem cell line via stereotactic injection in patients 2–13 months post‐stroke.^[^ ^120^, ^121^ ^]^ A larger sham‐controlled phase 2b study (PISCES‐III) was initiated in patients 6–12 months after stroke but was terminated early, without results.^[^ ^122^ ^]^

Taken together, clinical trials demonstrate that while safety has been consistently established, defining the optimal therapeutic window remains the central challenge, and significant work is still required to translate cell therapies into effective treatments for stroke.

## 6 Sex and Cell Therapies for Stroke

Sex influences the pathobiological processes and recovery trajectories following human stroke. The incidence and adverse outcomes of stroke rise in post‐menopause women, attributed to loss of estrogen‐dependent endothelial and platelet protection.^[^ ^123^ ^]^ Sex‐dependent difference in, acute cerebrovascular responses have been reported in a mouse model of photothrombotic stroke.^[^ ^124^ ^]^ Female mice exhibit an immediate cerebral blood flow drop by 40% within 5 min, while male only exhibit a 10% drop, following a gradual decrease to a comparable level to female by 48 h. However, the lesion size appears similar between male and female. The observed cerebral blood flow outcomes are at least partially due to sex differences in RhoA‐ROCK signaling and platelet activity.^[^ ^125^ ^]^ In spite of these biologic inference, human stem/cell‐therapy stroke trials, including recent large trials (MASTERS, TREASURE), do not report sex‐stratified outcomes.^[^ ^111^, ^126^ ^]^ Preclinical studies that compare recipient sex observe similar structural and functional benefits between male and female rodents. For example, Intravenous human umbilical cord blood‐derived MSCs or autologous BM‐MNCs, delivered at 24 h post‐stroke, improved motor/sensory recovery and reduces infarct volumes to the same extent in male and female rats after MCAO.^[^ ^127^, ^128^ ^]^ Similarly, in neonatal rats with hypoxic–ischemic brain injury, intraventricular transplantation of human neural stem cells, at 3 days following injury, was equally reparative in both male and female recipients.^[^ ^129^ ^]^ However, based on a meta‐analysis of 141 research articles on BM‐MSCs in preclinical ischemic stroke, female animals gained significant more benefits in sensorimotor recovery from cell treatment, as compared with male.^[^ ^130^ ^]^ Beyond recipient sex, donor‐cell sex may also have an effect. For neural progenitors, sex‐mismatched grafts (male NPCs to female host mice) in spinal cord injury enhanced lesional vascularization and cytotoxic T‐cell infiltration, without altering graft differentiation or axon growth, suggesting that donor cell sex can alter host responses to neural grafts.^[^ ^131^ ^]^ Of note, both MSCs and hPSCs exhibit sex‐dependent differences. For MSCs, emerging human data and systematic reviews indicate that sex impacts the proliferation, multi‐lineage differentiation capacity, and immunomodulatory potency.^[^ ^132^, ^133^ ^]^ Furthermore, sex‐linked transcriptomic divergence is reported during neural differentiation of human embryonic stem cells,^[^ ^134^ ^]^ and X‐chromosome inactivation erosion, a common feature of female hiPSCs, can modify gene expression that carry into neural derivatives.^[^ ^135^ ^]^ These data support donor sex as a source of functional heterogeneity of stem cell therapy. Finally, the optimal window for stem cell therapies might shift between males and females, and this constitutes an empiric question that future studies should test with a sex‐stratified design and endpoints.

## 7 Outlook: Reframing “Time is Brain”

For recanalization therapies, “time is brain” reflects the urgency of minutes and hours, as every delay costs neurons and can severely affect long‐term recovery. For regenerative cell‐based therapies, however, the clock runs differently. Here, timing means synchronizing delivery with the host brain's evolving receptivity, introducing cells when inflammation has subsided enough to allow survival, yet before plasticity and tissue potential are lost (Figure 2). Importantly, this window is unlikely to be universal: it may differ depending on the type of cell therapy, the delivery route, and the biological state of the patient. Identifying biomarkers to define the optimal “window of receptivity” will be essential, as this window is likely to vary across patients, opening once inflammation has subsided enough to permit graft survival, yet before plasticity and viable tissue potential are lost.

A key observation in literature is non‐standard, or imprecise, reporting of timing categories (hyperacute, acute, subacute, chronic), constituting a barrier for between‐study comparison, meta‐analysis, and compromising translatability. Therefore, we suggest a harmonized reporting framework for preclinical cell therapy studies. First, rather than reporting on the exact time from stroke onset, biological phases should be determined by the blood and imaging biomarkers, in addition to standard clinical assessments, and reported. Second, a common nomenclature could be adopted for the outcomes and benefits. Developing such a standard is well beyond the scope of this perspective, and can be best advanced by the community process, such as STAIR or STEPS consortium,^[^ ^9^, ^136^, ^137^, ^138^ ^]^ or remains as an open challenge.

Here we propose a complementary framing: “timing is brain” for cell‐based therapies, but here the clock is defined by the evolving biological phases rather than the elapse of minutes and hours. Future clinical translation will depend on standardizing this “window of receptivity,” guided by biomarkers, cell‐type‐specific mechanisms, and integration with reperfusion and rehabilitation strategies.

## Conflict of Interest

The authors declare no conflict of interest.

## Supporting information



Supplemental Table 1

Supplemental Table 2

Supplemental Table 3

Supplemental Table 4
